# Experience of Managing Conjoined and Parasitic Twins from a Developing Country

**Published:** 2013-12-05

**Authors:** Gnassingbé Komla, Adjenou Komla, Simlawo Kpatekana, Andele Komlan A, Mama Wakatou A, Akakpo-Numado Komlatsè G, Tekou Hubert A

**Affiliations:** Pediatric Surgery Department, University of Lomé, Togo; Radiology and Imaging Department, University of Lomé, Togo; Pediatric Surgery Department, University of Lomé, Togo; Pediatric Surgery Department, University of Lomé, Togo; Pediatric Surgery Department, University of Lomé, Togo; Pediatric Surgery Department, University of Lomé, Togo; Pediatric Surgery Department, University of Lomé, Togo

**Dear Sir,**

The conjoined twins (CTs) are one of the most challenging conditions in pediatric surgery especially in developing countries.[1] In developed countries, antenatal diagnosis of CTs is possible at 12th week of gestation, however, in developing countries like Togo, CTs are often discovered at birth.[2] Some work on CTs has been done in Central and West Africa.[3-5] The problems of antenatal diagnoses, preoperative work up, optimum postoperative management, and ultimately poor survival was reported by all. Herein, we describe three cases of CTs that were managed a tour facility.

**Case 1**

Female CTs, born by cesarean section found joined at abdomen and chest. Prenatal diagnosis was not made. No obstetrical ultrasound was done as well. The father was 22 year old and the mother of 15 year age. Clinical examination noted that the twins were in bad general state with omphalothoracic attachment and an omphalocele. A computerized tomography scan performed showed sternal apposition, single liver, two hearts but common pericardium, and separate bowel loops. A surgical separation was decided and carried out four days later. Exploration revealed the presence of two separate hearts but contained in the same pericardium. Both hearts were morphologically normal without any anatomic malformation. There were also two lungs for each twin. However, the twins shared the same liver located in the omphalocele. The liver had a linear zone to imagine a longitudinal cleavage of two fused livers. We made the separation of the livers through the linear region and CTs were separated completely (Fig. 1). Both CTs died one after other during operation.

**Figure:**
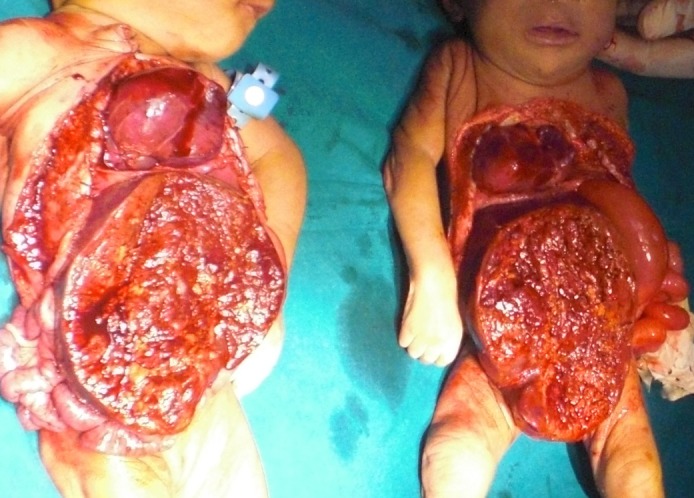
Figure 1: The separated twins (died)

**Case 2**

Male parasitic CTs were referred to us after 2 days of birth by cesarean section. The mother had three antenatal visits. No obstetrical ultrasound was performed. Clinical examination of the newborn showed normal upper and lower limbs, three limbs (two upper and one lower) were attached to epigastrium, and an omphalocele (Fig. 2). Echocardiography revealed fibrous mitral valves with minimal mitral regurgitation and a dilatation of right cavities and of the pulmonary artery, pulmonary hypertension and a small posterior pericardial detachment. Twenty-four hours later, the baby died from acute respiratory disorder without any fever.

**Figure:**
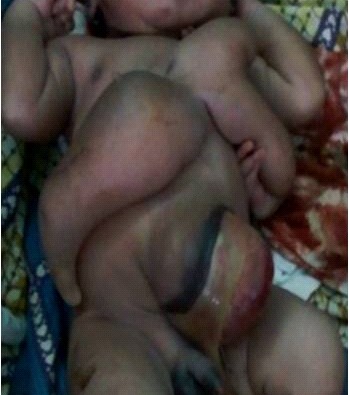
Figure 2: CTs with limbs attached to the epigastrium.

**Case 3**

CTs were born by cesarean section. Mother was of 22 year of age and the father of 26 year. No ultrasound was done during pregnancy. Clinical examination at birth revealed omphalopagus twins with single anus and a single umbilical cord (Fig. 3). Each twin had two upper limbs. However, there were only two legs. One of the twins had a systolic murmur. They died five days later.

**Figure:**
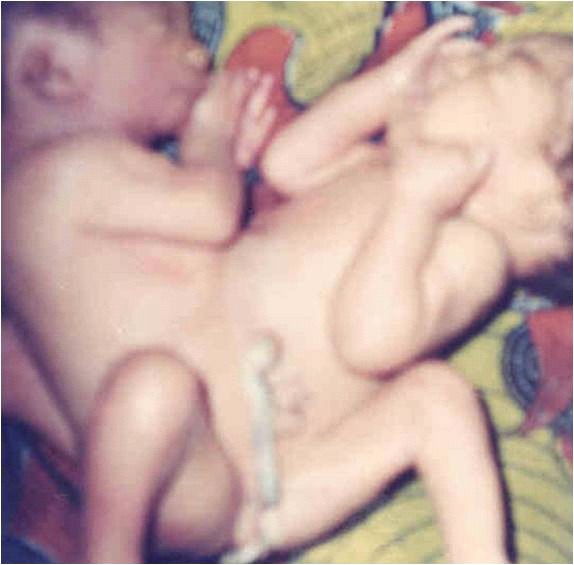
Figure 3: CTs twins with single anus and a single umbilical cord

Every aspect of management of CTs has problems in under-resourced and under-equipped centers. These problems are related to failure to have prenatal diagnosis, complications during labor and improper postnatal care. CTs are birth surprise resulting in chaos. In our cases, all pregnancies had to be converted to cesarean sections due to dystocia. Lack of anesthesiologist specialized in newborn surgery, and lack of a well-trained surgical staff are other areas that need improvement. Besides these factors associated major cardiac anomalies and fusion anatomy also determine the prognosis. Absence of preoperative diagnosis, inappropriate monitoring pre and per-surgery, and skills of team managing such cases lead to demise in all these cases.

## Footnotes

**Source of Support:** Nil

**Conflict of Interest:** None declared

